# Microseismic comprehensive evaluation method for coal burst: a case study in the Zhaolou Coal Mine

**DOI:** 10.1038/s41598-024-66294-5

**Published:** 2024-07-06

**Authors:** Heng Zhang, Liang Liang, Na Lin, Shuai Zhang, Miao-miao Cui, Zhong-gang Yang

**Affiliations:** 1 School of Civil Engineering, Nanyang Institute of Technology, Nanyang, 473004 Henan People’s Republic of China; 2 Henan International Joint Laboratory of Dynamics of Impact and Disaster of Engineering Structures, Nanyang Institute of Technology, Nanyang, 473004 Henan People’s Republic of China; 3 Shandong Tangkou Coal Industry Co., Ltd, Jining, 272000 Shandong People’s Republic of China; 4 Shandong Vocational College of Light Industry, Zibo, 255300 Shandong People’s Republic of China; 5 Binzhou Polytechnic, Binzhou, 256600 Shandong People’s Republic of China

**Keywords:** Coal burst, Microseism observation, Fissure development, Multiparameter, Time-varying, Engineering, Civil engineering

## Abstract

To explore the multiparameter precursor characteristics of pre- and post-coal burst. Based on a coal burst of LW 1305 in the Zhaolou Coal Mine, an early warning method combining stress‒strain curve and microseismic multiparameter is proposed. The research results show that coal burst was induced by the intrinsic static high-stress concentration and the strong external impact loading generated by fracturing of the key stratum. The precursors mainly characterize the enhancement trend of the *S* value, the sudden and sharp increase in the *A(t)* value, the continuous and abnormal decrease in the *b* value, the increasing absolute value of *Z* sharply and larger than 2, the continuous and abnormal decrease in the *Qt* value, and the dominant frequency moving to the low-frequency band. Essentially, many micro-fissures inside the key stratum initiated, converged and connected to form macro-fractures, which was verified by the attenuation rate of the *K* value. Considering the time-varying effect of the overlying stratum movement, the curves of the six parameters agree well with those of stress vs. strain, which indicates that it is reasonable to take the observed zone as a whole system to investigate the variation in the multiple parameters and fracturing of the key stratum. The research results can be applied to the monitoring, early warning and control of coal burst so that effective safety measures can be taken in real time.

## Introduction

Coal burst is a dynamic phenomenon of coal and rock masses in underground coal mining. When the mechanical system of coal and rock masses reaches the ultimate strength, the accumulated elastic energy is released suddenly and violently, resulting in roadway damage and casualties^1,2^. With the depletion of coal resources, the development of deep coal mining and complex mining conditions will obviously aggravate coal burst risk. Therefore, a comprehensive prediction and prevention system with multiple parameters should be established to monitor and forecast coal burst.

Many researchers have investigated multiple parameters before coal burst or great earthquakes. As pointed out by Li^3^, coal burst is among the most catastrophic dynamic hazards in coal mining. Since microseismic (MS) events are approaching coal burst, the location of coal burst can be accurately predicted^4,5^. In 1940, the mining bureau of the USA utilized the MS monitoring method with audible frequency and energy to forecast coal burst in hard rock coal mines^6^. Seismicity varied abnormally provided good evidence that roof fracturing could induce coal burst^7,8^. The abnormally high total fault area and the absolute value of Z, which is a precursor of earthquakes, can predict moderately strong earthquakes^9,10^. The reason why the MS activity of the fault was so rarely observed may simply be that the events were too small to be detected by a conventional seismic network, although other possibilities, such as a large critical size on mature faults, were not excluded^11^. Other researchers have concluded that larger earthquakes do not occur after excessive earthquakes and that larger earthquakes occur after absent earthquakes; moreover, they noted that the method of predicting the absence of earthquakes could have certain significance^12,13^. In addition, the MS activity and total fault area could be used to research the precursors of coal and gas outbursts^14^. According to the comprehensive analysis of rock mass classification, rock strength criterion, and 2D and 3D numerical simulation, Alber and Fritschen^15^ predicted MS events from the perspective of seismology.

In addition, acoustic emission (AE) has been studied by several researchers. The AE activity associated with rock failure and friction was studied in combination with rock information by Zhang et al.^16^, Mansurov^17^, Rudajev^18^ and Makoto et al.^19^. Considering strain, dissipation and kinetic energies as well as the work of external force, a variation principle was established by Gorgogianni and Papargyri-Beskou^20^ to analyze the dynamics of fissured poro-elastic rocks. Based on the shallow buried coal seams covered with thick loose layers in hilly loess areas of western China, according to many researchers^21–23^, laboratory and in situ evidence has shown that under the superposition of static and dynamic loads, the original minor fissures in coal and rock masses initiate, converge and connect into macro-fractures, eventually leading to the failure of coal and rock masses. In addition, many other scholars have illustrated the source mechanism and failure mechanism of coal burst in detail and noted that the coal burst source and failure source may not be the same for different types of sources and failure mechanisms^24,25^. The mine earthquakes were divided into two types by Gibowiczs and Kijkoa^6^: one was related to the rupture and deformation of the mining face, and the other was related to the movement of large geological sections, such as faults. Horner and Hasegawa^26^ indicated that most mine earthquakes involved shear ruptures related to the structure. Zhang et al.^27^ proposed a method for hazard assessment in coal mines based on the seismic energy distribution. Several laboratory and numerical simulation studies have been performed on rock structure failure^28,29^. Dou and Drzezla^30^ modified the complex method of determining the hazard state of rock bumps in hard coal mines.

Generally speaking, the MS activity, multiple parameters, stress, deformation and failure laws have been determined, and the conclusions have been confirmed by the majority of scholars. However, the MS multiparameter precursory characteristics of coal burst have not been well researched and are less well documented than coal burst mechanisms and seismicity predictions. Moreover, for coal burst induced by static high-stress concentrations during the beginning of mining, MS precursors combined with fissure development have not been further studied. In particular, considering the involvement of shock waves generated by key stratum fracturing, the triggering mechanism and warning methods for coal burst have rarely been investigated and reported. Therefore, understanding MS precursors combined with fissure development has been a central problem in monitoring and prewarning coal burst. In this paper, a coal burst hazard that occurred at the Zhaolou Coal Mine with strong coal burst risk was investigated in detail by the MS system. Based on the analysis of the MS results, the multiparameter precursor characteristics combined with the fissure development rules pre- and post-coal burst were revealed and presented as effective prewarning criteria.

## Stress‒strain curve and multiple MS parameters

### Stress‒strain curve

**Figure 1:**
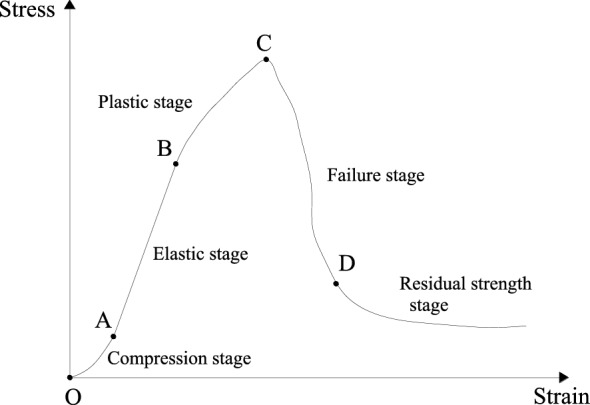
The stress–strain curves.

Based on the stress–strain curve characteristics of rock mass failure under uniaxial compression^31,32^, the general complete stress‒strain curve is divided into five stages, as shown in Fig. 1. The OA section is defined as the compaction stage, caused by micro-fissure closure under compression; the AB section is defined as the elastic stage, where the relationship curve of stress and strain is a straight line; the BC section is defined as the plastic stage, caused by some microfractures emerging and forming strictly parallel to the load axis direction; the CD section is defined as the failure stage, in which the microfractures gradually connect into the macro-fractures and finally slip along the macro-fractures; and the last section is defined as the residual strength stage. However, in deep coal mines, the stress is high in coal and rock masses, and the residual strength stage can be ignored when studying coal burst. For the sake of simplification, we studied only sections AB, BC, and CD in a deep coal mine.

### MS activity *S* value

The MS activity S value contains MS activity in time, space and magnitude, which are as follows: event count, average MS magnitude, maximum MS magnitude, and the concentration of the MS distribution and its memory effects^33^. It is expressed as follows:

1 $$ S = 0.117\lg (N + 1) + 0.029\lg \frac{1}{N}\sum\limits_{i = 1}^{N} {10^{{1.5 \times M_{i} }} } + 0.015 \times M $$

where *N* is the total event count, *M*_*i*_ is the MS magnitude, and *M* is the maximum MS magnitude. Commonly, a large energy MS occurs after the increase in the S value.

### Fault total area $A(t)$ value

When evaluating the activity level of MS using energy and event counts, it is important to note that the number of low-energy events is usually much greater than that of high-energy events. This means that the event count is predominantly determined by low-energy events, while high-energy events are usually responsible for the total energy, thereby ignoring low-energy events. To address this discrepancy, the total defect area A(t) has been defined, which takes into consideration both the number of events and the energy^33,34^.

2 $$ A(t) = \sum\nolimits_{{k = k_{0} }}^{k - 1} {N\left( k \right)} \cdot 4.5^{{k - k_{0} }} $$

where $k_{0}$ is the lower limit of the statistical MS magnitude and $k$ is the magnitude of each event. $N\left( k \right)$ is the event count of MS magnitude $k$ (correspondingly, the energy is 10^ k^ − 10^ k+1^ J).

Strong MS events tend to occur in fault zones, and a large amount of energy accumulated in the seismogenic process is released through fissure development. Therefore, the greater the quantity of fissures in the fault zone is, the greater the possibility of strong MS occurrence. Theoretically, before high-energy release occurs, $A(t)$ commonly manifests an abnormally high value, which indicates that MS activity will obviously increase.

### *Z* value

The average magnitude of MS event samples $\overline{{m_{j} }}$ in the observation zone is defined as

3 $$ \overline{{m_{j} }} = \frac{1}{k}\sum\limits_{i = 1}^{k} {m_{i} } $$

where $m_{i}$ is the magnitude of each event. The Z value is defined as

4 $$ Z = \frac{{\overline{M} - \overline{m} }}{{\sqrt {\frac{{\sigma_{M}^{2} }}{N}} + \frac{{\sigma_{m}^{2} }}{n}}} $$

where $\overline{M}$ is the arithmetic mean of all average magnitude samples $\overline{{m_{j} }}$ in the entire monitoring period (a relatively stable amount to manifest background characteristics of the observation area), $\overline{m}$ is the arithmetic mean of the average magnitude samples $\overline{{m_{j} }}$ in the observation period, N and n are the quantities of $\overline{M}$ and $\overline{m}$ samples, respectively, and $\sigma_{M}^{{}}$ and $\sigma_{m}^{{}}$ are their standard deviations^33,34^.

Since the Z value approximately follows the standard normal distribution, it has the significant characteristics of a normal distribution. Specifically, when Z = 1.64, the result is significant at the 90% level. When Z = 1.96, the result is significant at the 95% level. When Z = 2.57, it has a significance level of 99%. Accordingly, the variation in the average size of samples can reflect the significant characteristics of MS activity. When Z = 0, the rate of MS event occurrence is the same as that in the background condition. If Z < 0, the probability will increase. If Z > 0, the probability will decrease. Regardless of whether Z > 2 or Z < 2, both are low-probability events, and the occurrence of high-energy MS events is the same. Therefore, the absolute value of Z can be defined as an abnormal critical value larger than 2. The larger the absolute value of Z is, the greater the risk level of coal burst.

### Lack of shock b-values

According to the Utsu Tokuji formula^35,36^, the b value in the Gutenberg formula can be defined as

5 $$ b = \frac{0.4343}{{\overline{M} - M_{0} }} $$

6 $$ \overline{M} = \sum\limits_{i = 1}^{N} {\frac{{m_{i} }}{N}} $$

where $\overline{M}$ is the average level of MS energy in the statistical period, $M_{0}$ is the starting level, and N is the total number of MS events. According to Eq. (7), the variation in the b value depends on the average magnitude $\overline{M}$. Under conditions of normal MS activity, the b value in an area is stable, and $\overline{M}$ should also be relatively stable, which indicates that $\overline{M}$ can describe the average level of MS activity in an observation zone. If the short-term average magnitude $\overline{{m_{i} }}$ is smaller than the long-term average magnitude $\overline{M}$, some high-energy events will likely occur in the zone to compensate for the long-term average magnitude, which is the basic definition of the lack of a shock b value. If the short-term average magnitude $\overline{{m_{i} }}$ is larger than the long-term average magnitude $\overline{M}$, high-energy MS events will not occur in this zone. Therefore, if the b value in the statistical zone is abnormally low, a high-energy MS event is extremely likely to occur.

### MS entropy *Q*_*t*_ value

Considering the preparatory occurrence and development process of earthquakes as an open system, given the basic ideas of the theory of the dissipative structure, the MS entropy is introduced as an order parameter, describing the scale of order associated with the earthquake distribution in time^35^. The evolution of seismic activity before and after a large earthquake has been studied by using the MS entropy *Q*_*t*_ value. The preliminary results show that the MS entropy *Q*_*t*_ decreases in certain regions as large earthquakes approach. This means that the distribution of earthquakes gradually changes in order of disorder. Similarly, when the stress field reaches a certain value, the MS distribution over time gradually changes in order of disorder, that is, the MS entropy *Q*_*t*_ increases. The MS entropy *Q*_*t*_ value can be defined as

7 $$ Q_{t} = \frac{{ - (1/n)\sum\limits_{i = 1}^{n} {p_{i} \ln p_{i} } }}{\ln (n - 1)} $$

where *n* is the total event count in a certain time window and $p_{i}$ can be defined as

8 $$ p_{i} = \frac{{t_{i + 1} - t_{i} }}{{t_{n} - t_{1} }} $$

where $t_{i}$ is the occurrence time of the $i$ MS event, and the $p_{i}$ value ranges from 0 to 1.

The MS entropy *Q*_*t*_ value mainly describes the cluster features of the MS event distribution over time and the process from disordering to ordering over time. In theory, in the precursory process of high-energy MS, the MS entropy *Q*_*t*_ decreases. Essentially, the MS event distribution increases in an orderly manner over time. Before high-energy MS, micro-disorder cracks develop toward macro-order cracks, and the MS entropy *Q*_*t*_ decreases significantly, which is similar to seismological entropy. Therefore, we can take the decreasing MS entropy *Q*_*t*_ value as a precursory signal of coal burst.

### Dominant frequency

The MS signals mainly record the mining tremors. It is important for us to forecast the dynamic danger of a mine (such as coal burst) by explaining, analyzing and making use of the recorded information, especially via spectrum analysis. In general, MS is the dynamic phenomenon of stress and strain in coal and rock masses caused by mining.

The basic principle of the Fourier transform is to assume that in some periods, the initial signal $f(t)$ fits the Dirichlet condition: (1) if there are discontinuities, then the number should be countable; (2) in a single period, the signal contains a limited number of maximum and minimum points; and (3) the signal can be effectively integrated during this period. Then, signal $f(t)$ can be represented by the superposition of different sinusoidal functions; that is, the specific expression of the Fourier transform is as follows^37^:

9 $$ F(w) = \int_{ - \infty }^{ + \infty } {f(t)} e^{ - jwt} dt $$

where $e^{jwt}$ represents the base of the Fourier transform. Then, the inverse Fourier transform is:

10 $$ f(t) = \frac{1}{2\pi }\int_{ - \infty }^{ + \infty } {F(w)} e^{jwt} dw $$

When coal and rock masses develop fissures that connect, the dominant frequency of MS waves shifts. The dominant frequency of MS reaches its lowest point prior to the failure of coal and rock, despite the low number of recorded events. This implies increased total energy and a greater likelihood of a high-energy MS event occurring. A significant decline in MS frequency from hybrid to low-frequency serves as a compelling indication of an impending failure of coal and rock bursting.

## Basic site description of LW1305

### Production and geological conditions of LW 1305

Coal burst occurred in the 1305 island face with longwall fully mechanized mining. It was located in the No. 1 mining area at the Zhaolou Coal Mine, the east side was the track dip in the No. 1 district, the west side was the boundary of the No. 1 mining area, the north side was adjacent to the goafs of LW 1304, LW 1303 and LW 1302, and the south side was the goafs of LW 1306 and LW 1307. The selected LW 1305 was an island face. LW 1305 was 574 m in the strike direction and 137 m in the dip direction, the recoverable reserves were 54.8 million tons, and the maximum mining depth was 998 m. The mining seam was the #3 coal seam, whose thickness varied from 2.8 to 9.0 m (the average is 6.1 m), the average dip angle was 8°, and the average Protodyakonov coefficient was 1.6. According to the borehole dataset, the immediate roof of LW 1305 was mudstone and siltstone with a thickness of 1.2–8.4 m, the main roof was hard and steady medium sandstone with a thickness of 4.5–20.4 m, the immediate floor was mudstone with a thickness of 1.5–3 m, and the primary floor was fine sandstone with a thickness of 6.4–11.8 m.

There was a splitting region of the #3 coal seam in the vicinity of the open-cutting LW 1305 coal seam, the thickness of the upper #3 coal seam was 1.0–1.7 m, the thickness of the lower #3 coal seam was 2.7–6.4 m, the splitting spacing was 0.7–14.6 m, and the western region of the open-off cut in LW 1305 was the noncoal district with igneous rock intrusion. The abnormal deposition of the lower 3# coal seam led to the thinning of the coal seam.

**Figure 2:**
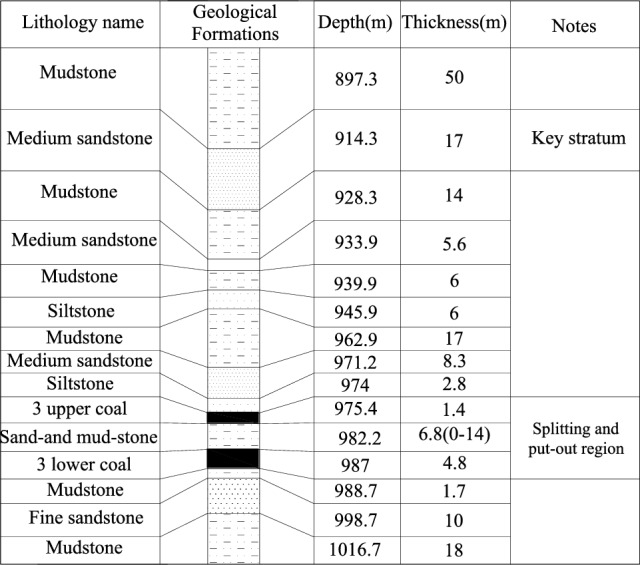
Comprehensive column illustration of the coal and rock layers in LW 1305.

**Figure 3:**
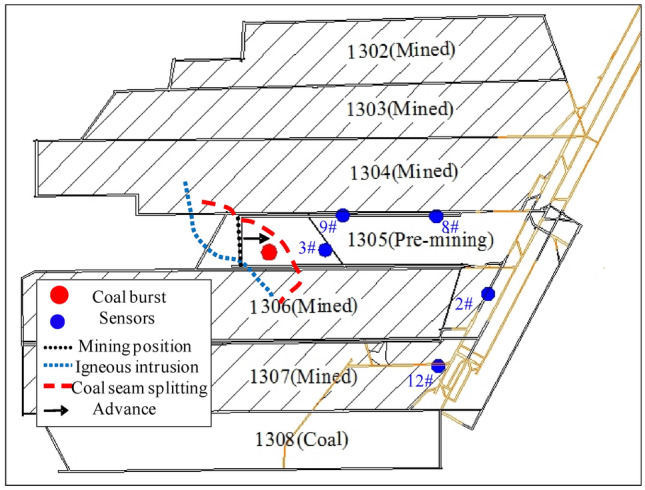
Entry layout, burst source distribution, and disposition of the geophone stations in LW 1305.

Figure 2 shows a comprehensive column illustration of the coal and rock layers. Figure 3 shows the plane sketch of LW 1305. Until the coal burst occurred, the advancing distance in the headentry was 7.2 m, and the advancing distance in the tailentry was 1.6 m.

### MS monitoring system

The seismological observation system (SOS) MS monitoring system developed by the Poland Central Mining Institute was installed at the Zhaolou Coal Mine on December 22, 2010, and mainly consisted of a real-time monitoring recorder, analyzer, sensors and digital transmission system^34^. The single vertical-component sensor has a frequency range of 1–600 Hz, with a horizontal location error of less than 20 m and a vertical positioning error of less than 30 m. The sampling rate is 500 Hz, and the A/D converter is 16 bits. The maximum data transmission rate is 1 MB/s. The maximum data transmission distance is 10 km. The system can continuously and automatically collect and filter MS signals, accurately calculate the occurrence time, released energy, and three-dimensional coordinates of shock events using the Powell location algorithm. The location model proposed is a constant velocity model calibrated by the arrival time residual error of several high-energy shock signals. The sensor is of the broadband and moving-coil type, with a resonance frequency of 4.5 ± 0.75 Hz. Cable noise can be eliminated by a 50 Hz bandpass filter controlled by a switch. The system records events using a triggering mode. An MS event is only recorded and accurately located when more than four sensors simultaneously receive a clear waveform. The following study is based on MS signals monitored by the abovementioned SOS.

### Sensor arrangement

Sixteen sensors installed in the underground roadways were used for the three-dimensional monitoring of the MS system. LW 1305 was surrounded by a total of 7 sensors. According to the mining position on July 29, 2015, and the MS signals, 5 sensors (#2, #3, #8, #9, and #12) closest to LW 1305 were chosen for analysis (in Fig. 3). Table 1 shows the three-dimensional coordinates of the 5 sensors. The elevation of LW 1305 ranged from − 963 to − 828 m, and the headentry and tailentry were coal roadways.

**Table 1:** Three-dimensional coordinates of 5 sensors.

Sensors number	x/m	y/m	z/m
2	20,403,945	3,917,106	− 935
3	20,403,490	3,916,980	− 961
8	20,403,660	3,917,233	− 928
9	20,403,449	3,917,119	− 949
12	20,403,903	3,916,874	− 945

### Coal burst occurrence

**Figure 4:**
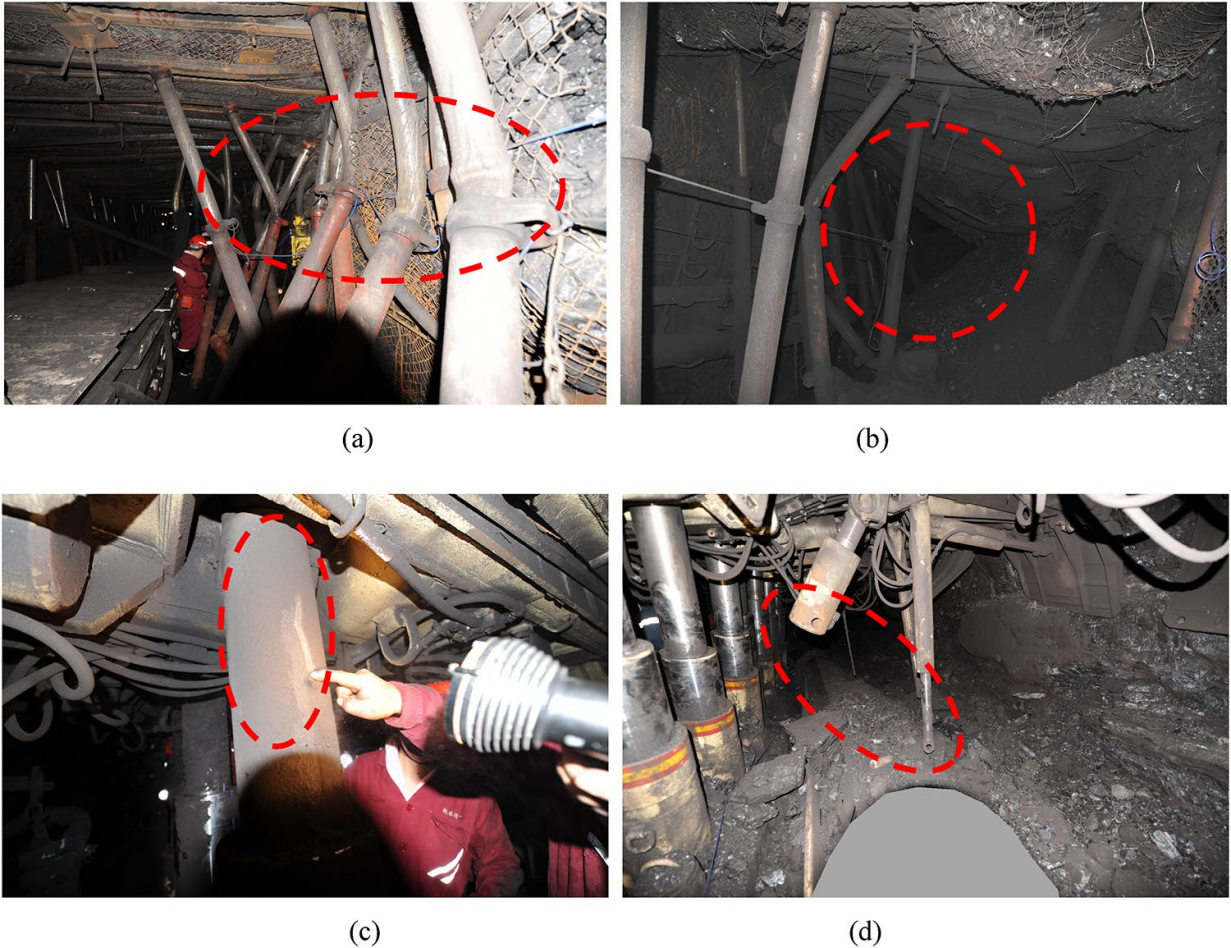
Photographs of coal burst damage in roadways and working faces. (**a**) Many single propps were bent by shock pressure on the headentry. (**b**) Large deformation on part of the tail entry. (**c**) A large amount of coal dust was ejected on the hydraulic support props. (**d**) The pins were cut off.

At 02:45:34 on July 29, 2015, coal burst occurred in LW 1305, and the energy detected by SOS MS monitoring was 2.5 × 10^6^ J. The three-dimensional coordinates (x, y, and z) of the source were 20,403,340 m, 3916,935 m, and − 872 m, respectively. Based on the source distribution in the z-direction, it can be verified that coal burst was associated with fracturing of the key fine sandstone strata overlying the #3 coal seam. Before the coal burst occurred, the coal mining machine cut into the coal, and LW 1305 suddenly underwent continuous coal blasting. After the coal burst, the upward and downward exits of the working face were almost completely blocked by the heaving coal materials in the tail entry and head entry, and only a small amount of breeze circulated throughout the working face. Figure 4 shows photographs of coal burst damage in the roadway and working face.

**Figure 5:**
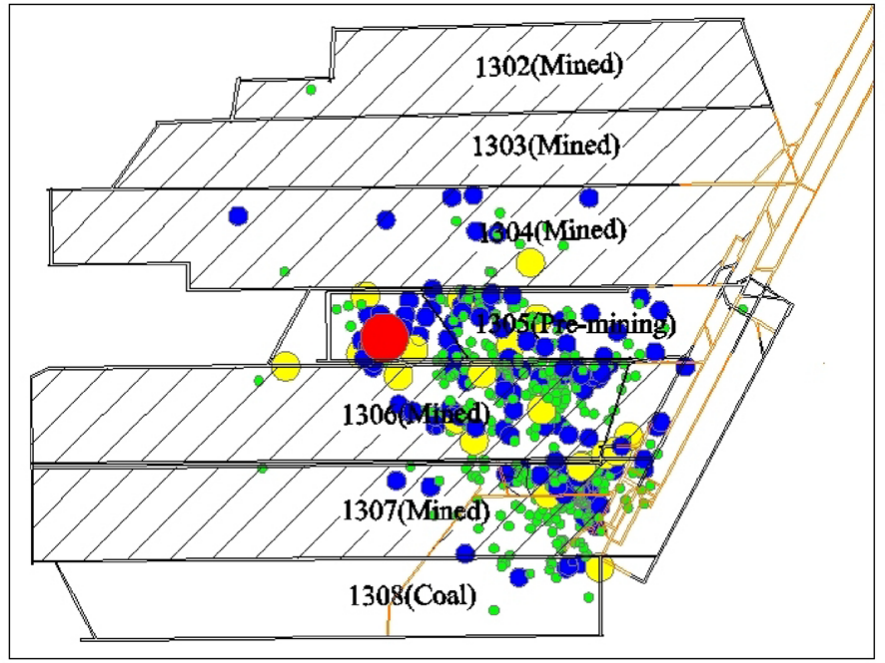
The MS source location evolution with different energy ranges. Notes: Green: 0–10^3^ J; Blue: 10^3^–10^4^ J; Yellow: 10^4^–10^5^ J; Red: > 10^5^ J.

LW 1305 is a deep island face, and there was igneous rock intrusion, splitting and pinching out of coal seams in the vicinity of the region damaged by coal burst (Figs. 2 and 3). Static high-stress concentrations easily formed, and the majority of the sources were located in the vicinity of the region, which indicated that the static and tectonic stresses were strongly concentrated in the region (Fig. 5). In addition, the width of LW 1305 is 137 m, which is equivalent to a large coal pillar, and it inevitably generates a high stress concentration and accumulates a large amount of elastic energy inside the large coal pillar, which significantly exacerbates the stress concentration level. The external triggering factor of the coal burst was fracturing of the key fine sandstone stratum associated with the mining. Ultimately, the severe static high-stress concentration, especially combined with the strong impact loading generated by fracturing of the key stratum, induced the disastrous coal burst.

## Analysis of MS monitoring results

In this paper, we analyzed the multiparameter precursors, frequency spectra and coda waves of MS signals combined with the general complete stress‒strain curve of rock to investigate the warning criteria for coal burst. MS multi-parameter was slip-sweep analyzed with 5 days window, 1 day time interval. Figure 5 shows the MS source distribution from July 10, 2015, to August 15, 2015, in LW 1305.

### Variation in the S value

**Figure 6:**
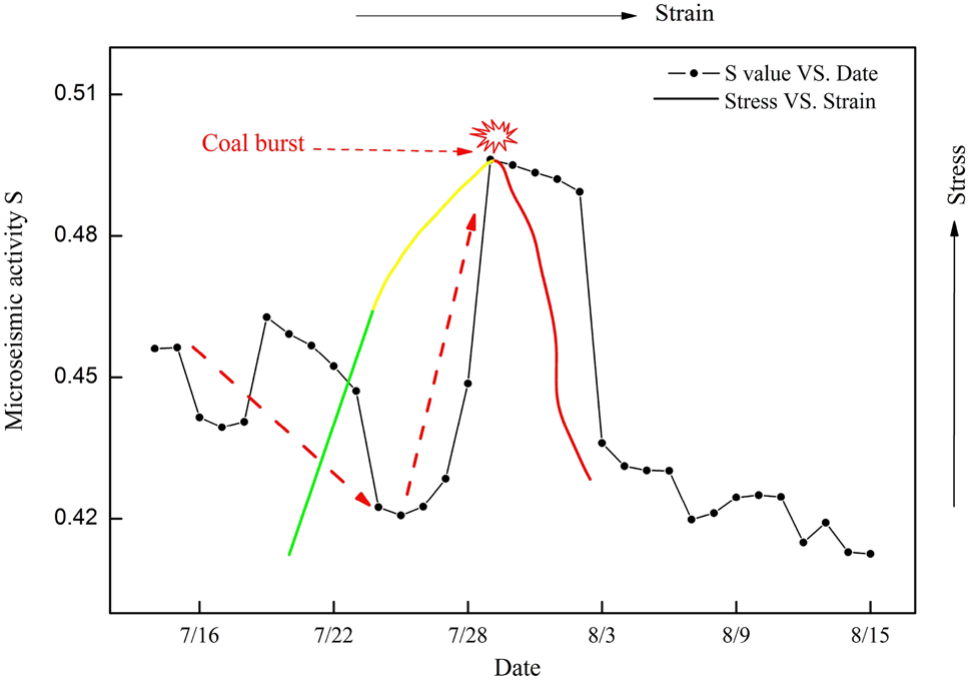
Variation curves of the MS activity S value and stress versus strain.

Figure 6 shows the curve of the S value from July 15, 2015, to August 15, 2015, in LW 1305. According to Fig. 6, before July 24, the S value basically decreased from 0.46 to 0.42, which indicated that LW 1305 and the key stratum were accumulating elastic energy, corresponding to the elastic stage in the curve of stress vs. strain, and the individual growing S value (from July 19 to 23) was associated with roof caving in LW 1307. However, from July 25 to July 29, the S value generally and sharply increased by 20%, which indicated that many micro-fissures inside LW 1305 and the key stratum initiated, converged and connected to form macro-fractures, corresponding to the plastic stage in the curve of stress vs. strain. On July 29, the S value reached a peak value of 0.50. After that, the S value suddenly decreased from 0.50 to 0.40, corresponding to the failure stage in the curve of stress vs. strain. According to formula (1), the S value was a comprehensive weight. Therefore, the enhancement trend of the S value can be regarded as an effective precursory sign for warning of coal burst risk.

### Variation in the total fault area $A(t)$

**Figure 7:**
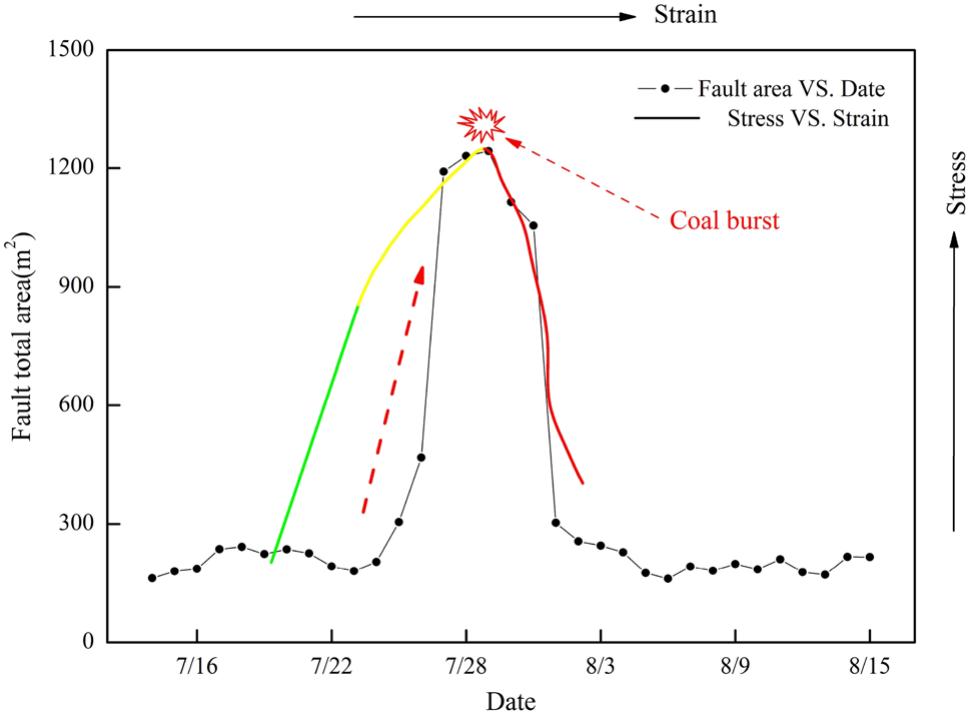
Variation curves of the total fault area and stress versus strain. Note: the green line represents the elastic stage, the yellow line represents the plastic stage, and the red line represents the failure stage.

Figure 7 shows the variation curve of the total fault area for MS events from July 15, 2015, to August 15, 2015, in LW 1305. Before July 23, the total fault area was generally lower and basically remained stable at 200 m^2^, even though it slightly decreased. However, from July 23 to July 29, the total fault area generally and sharply increased by 525%. When coal burst was induced by fracturing of the key stratum, the total fault area reached a maximum at 1250 m^2^. After that, the value significantly decreased to 200 m^2^. Therefore, the sudden and sharp increase in the total fault area can be regarded as a precursory warning of coal burst risk. The larger the total fault area was, the higher the coal burst risk level was.

### Variation in the Z value

**Figure 8:**
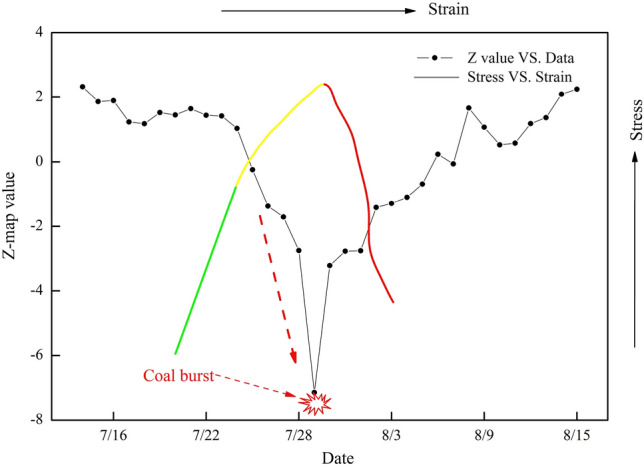
Variation curves of the Z value and stress versus strain. Note: the green line represents the elastic stage, the yellow line represents the plastic stage, and the red line represents the failure stage.

Figure 8 shows the Z value variation curve from July 15, 2015, to August 15, 2015, in LW 1305. It can be seen from Fig. 8 that before July 23, the Z value basically remained stable at 1.5, and its absolute value was basically less than 2. However, from July 23 to July 29, the Z value suddenly and sharply decreased. Especially prior to the coal burst (from July 27 to July 29), its absolute value was much larger than 2 and reached a maximum (7.3) on July 29, which indicated that the danger level of the coal burst at this stage was very high. Ultimately, the coal burst was triggered on July 29. After that, the absolute value of Z significantly decreased below 2 on August 2. Therefore, the phenomenon in which the absolute value of Z sharply increased and was greater than 2 can be regarded as an effective precursor for evaluating coal burst risk.

### Variation in the b value

**Figure 9:**
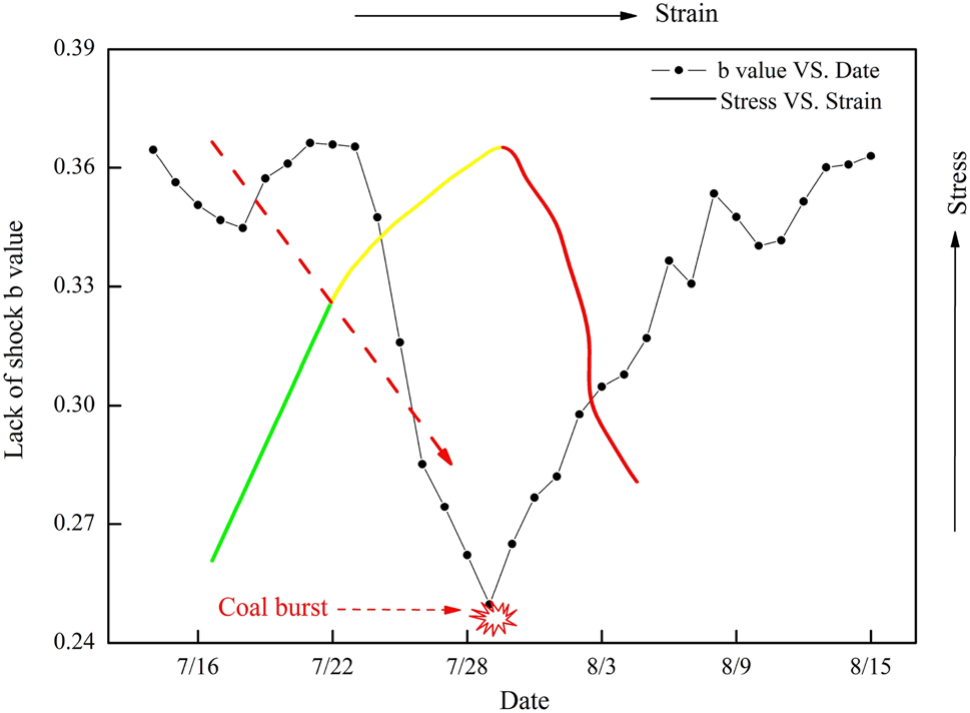
Variation curves of the b value and stress versus strain. Note: the green line represents the elastic stage, the yellow line represents the plastic stage, and the red line represents the failure stage.

Figure 9 shows the b value variation curve from July 15, 2015, to August 15, 2015, in LW 1305. As shown in Fig. 9, before July 29, the b value basically declined, and the individual increase in b value was associated with roof caving in LW 1307. Especially from July 23 to July 29, the b value rapidly and abnormally decreased, which indicated that some higher-energy events would occur to compensate for the average magnitude due to the insufficient increase in MS energy. On July 29, coal burst was inevitably triggered, with a b value of 0.25. After that, the b value began to rise to a higher level. Therefore, it can be verified that continuous and abnormally low b values may be regarded as effective precursors of coal burst risk.

### Variation in the $Q_{t}$ value

**Figure 10:**
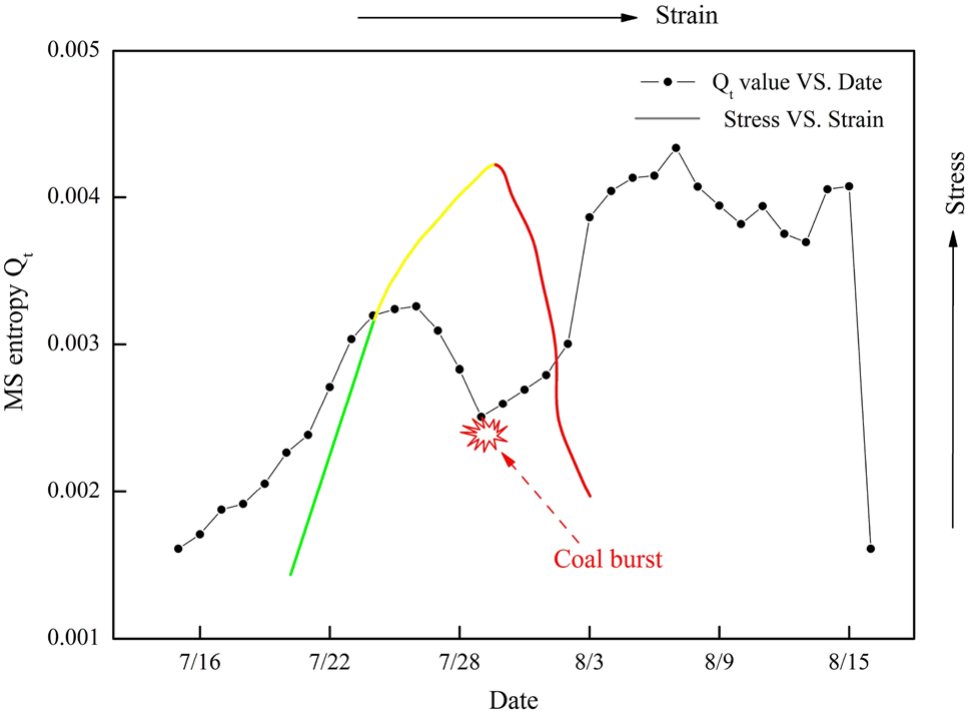
Variation curves of the MS entropy and stress versus strain. Note: the green line represents the elastic stage, the yellow line represents the plastic stage, and the red line represents the failure stage.

Figure 10 shows the $Q_{t}$ value variation curve from July 15, 2015, to August 15, 2015, in LW 1305. As shown in Fig. 10, before July 26, the $Q_{t}$ value gradually increased, which indicated that the MS activity in this period was normal and that the whole system in the observed region was free from or only slightly influenced by external factors. However, from July 26 to July 29, the $Q_{t}$ value significantly decreased, which indicated that the whole system in the observed region was remarkably and abnormally influenced by the static high stress, mining activity in LW 1305 and roof caving in LW 1307. On July 29, coal burst inevitably occurred. After that, the $Q_{t}$ value gradually increased. Therefore, it can be verified that the continuous and abnormal decrease in $Q_{t}$ may be regarded as an effective precursor of coal burst risk, and coal burst is likely to occur.

Interestingly, if the observed region was taken as a whole system, the variation in the $Q_{t}$ value would have a strong correlation with the curve of stress vs. strain. Similarly, according to the above analysis of the S value, $A(t)$ value, Z value, and b value, the variation in these multiple parameters was strongly correlated with the curve of stress vs. strain. Therefore, considering the time-varying effect of the stratum, it was reasonable to take the observed region as a whole system to research the variation in multiple parameters. However, the coal burst was immediately induced by fracturing of the key stratum. The fracturing of the key stratum should be a key problem for forecasting coal burst.

### Waveform and frequency-spectrum distribution of coal burst

**Figure 11:**
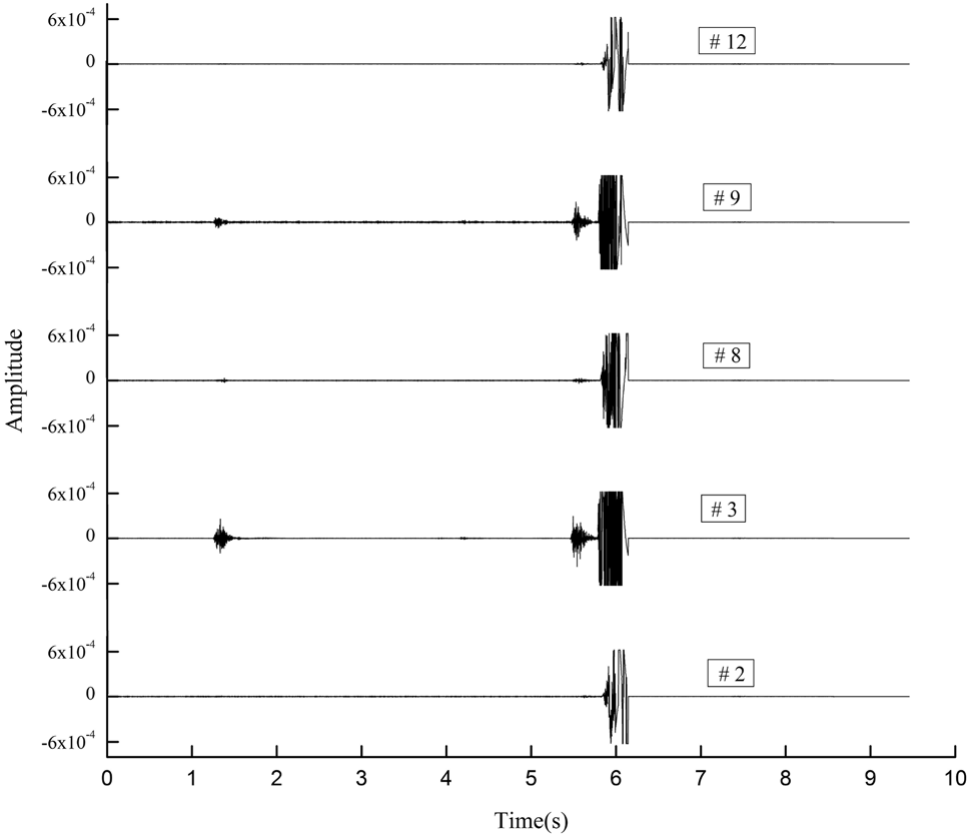
Original MS waveforms of coal burst recorded by sensors 2, 3, 8, 9, and 12.

**Figure 12:**
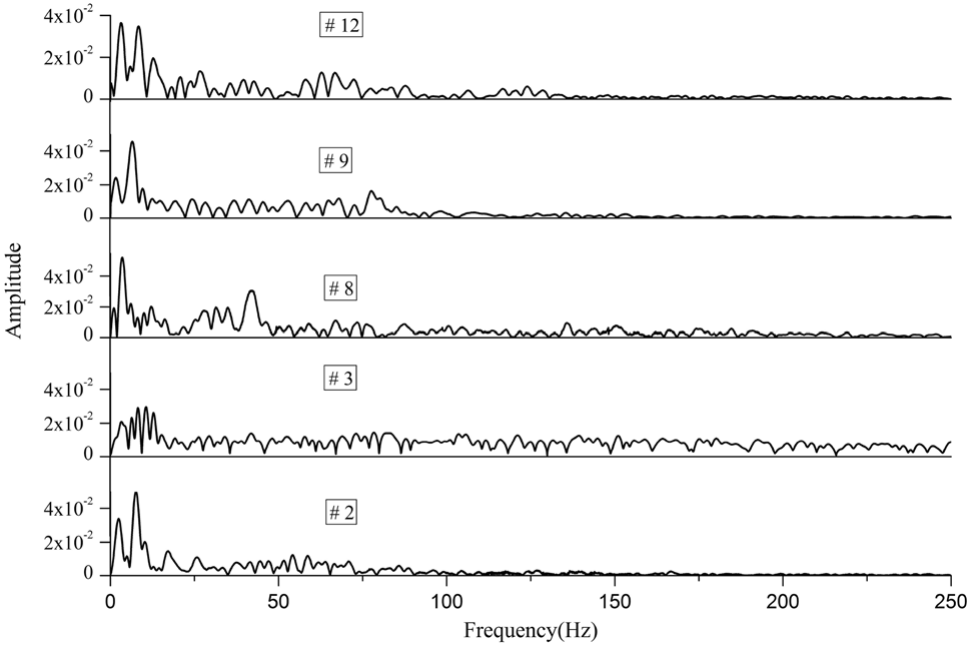
Frequency-spectrum distribution of coal burst recorded by sensors 2, 3, 8, 9, and 12.

Figure 11 shows the original MS waveforms of coal burst (namely, the signal produced by intensive fracturing of the key stratum) recorded by sensors 2, 3, 8, 9, and 12. With the same X-axis, multiple Y-axes were adopted to conveniently and comparatively study the waveforms. Figure 12 shows that the maximum vibration velocity of the coal burst signals reached 6.24 × 10^–4^ m/s, as recorded by 9 sensors, and five maximums of 2, 3, 8, 9, and 12 sensors were nearly equal, which demonstrated the intensive fracturing of the key stratum. All of the signal durations were less than 1 s. Before the coal burst signal reached the sensors, some low-amplitude signals were received by 3 and 9 sensors (as shown in Fig. 11), not by 2, 8, and 12 sensors, which were induced by micro-fissures. The signals recorded by sensors 3 and 9 were much stronger than those recorded by sensors 2, 8, and 12. Considering that the distances between the source and 2, 3, 8, 9, and 12 sensors were 632 m, 180 m, 441 m, 227 m, and 570 m, respectively, an attenuation effect was produced on the low-amplitude signals and the coal burst signals.

**Figure 13:**
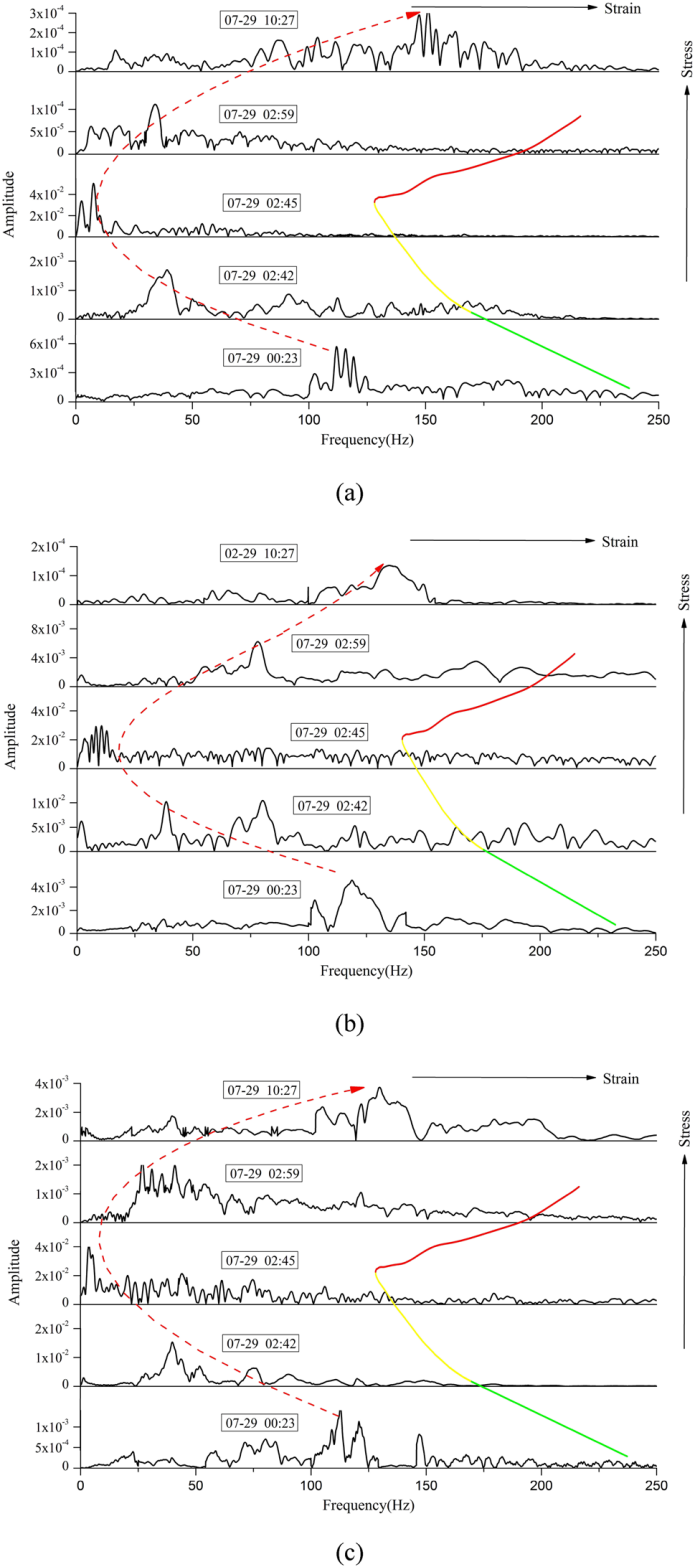
Evolution of the dominant frequency of MS signals before and after coal burst. (**a**) 2 sensors, (**b**) 3 sensors, (**c**) 9 sensors. Note: the green line represents the elastic stage, the yellow line represents the plastic stage, and the red line represents the failure stage.

Figure 12 shows the amplitude spectrum-frequency distribution curves of the coal burst signals recorded by sensors 2, 3, 8, 9, and 12. Similarly, for the same X-axis, multiple Y-axes were adopted to conveniently and comparatively study the spectrum frequency. As shown in Fig. 13, the dominant frequency ranges of the 2, 3, 8, 9, and 12 sensors were 1–13 Hz, 0.5–16 Hz, 1–14 Hz, 0.5–15 Hz, and 1–15 Hz, respectively, and the peak-spectrum frequencies were 7 Hz, 8 Hz, 7 Hz, 9 Hz, and 8 Hz, respectively. The lower dominant frequency indicated that many micro-fissures inside the key stratum initiated, converged and connected to form macro-fractures; then, fracturing of the key stratum occurred, and finally, coal burst was triggered. Based on the principle that the vertical interval can produce a significant attenuation effect on the shock wave and that the dominant frequency will obviously move to the low-frequency band (Lu et al. 2015), the dominant frequency ranges of sensors 2, 8, and 12 were obviously narrower than those of the other sensors.

For most MS signals, the low-frequency component is usually the most important component, which can manifest the main features of signals, while the high-frequency component is generally associated with noise and disturbances, and if the high-frequency part of the signal is removed carefully, the key signal characteristics can still be retained. Based on the characteristics of the SOS MS system, it can automatically filter shock signals, and coal burst signals do not require special handling (Lu et al. 2015). Considering shock wave propagation and attenuation laws, to reveal the frequency-spectrum characteristics of coal burst induced by roof caving, the signals recorded by the 2, 3, 8, 9, and 12 sensors closest to the source are analyzed in detail.

### Evolution of the dominant frequency

Figure 13 shows the evolution of the dominant frequency of the MS signals before and after the coal burst. The evolution of the dominant frequency is accurately analyzed. Based on the source event selection methods of Lu et al. and Dou et al.^37,38^, the chosen MS signals include two precursors, a mainshock, and two aftershocks, and their three-dimensional coordinates are almost the same. They were recorded at 00:23 on July 29, at 02:42 on July 29, at 02:45 on July 29, at 02:59 on July 29, and at 10:27 on July 29. Similarly, for the same X-axis, multiple Y-axes were adopted to conveniently and comparatively study the spectrum frequency. As shown in Fig. 13, prior to coal burst, the dominant frequency of precursor signals was in the high-frequency band and gradually moved to the low-frequency band, and the MS activity increased, which indicated that many micro-fissures inside the large coal pillar and key stratum initiated, converged and connected to form macro-fractures, corresponding to the plastic stage in the curve of stress vs. strain. When coal burst occurred, the dominant frequency of the mainshock signals clearly decreased to a minimum in the low-frequency band, which indicated that the failure type of the coal and rock mass was mainly macro-fracturing. After coal burst occurred, the dominant frequency of aftershock signals gradually shifted to the high-frequency band, which indicated that a large number of micro-fissures had formed and that the MS activity decreased, corresponding to the failure stage in the curve of stress vs. strain. Therefore, the phenomenon in which the dominant frequency of MS events moves from the high-frequency band to the low-frequency band can be verified as an effective index for warning of coal burst risk. The lower the dominant frequency was, the higher the coal burst risk level was. Similarly, the dominant frequency of fracturing of the key stratum was strongly correlated with the curve of stress vs. strain.

## Uncertainty and limitations

The data source of these evaluation methods is the SOS MS monitoring system, so the accuracy of the data has a great impact on the accuracy of the research results. At present, the SOS MS monitoring system is mainly based on the time difference between the P wave and S wave to determine the source location. However, P waves and S waves are affected by many factors in the propagation process, such as coal and rock strength, weak interfaces, internal fissures and density differences. These factors can affect the propagation speeds of P waves and S waves, resulting in inaccurate time differences and affecting the positioning accuracy. Moreover, the propagation energies of P waves and S waves are also affected by the above factors, resulting in inaccurate monitoring of source energy. However, fortunately, the MS activity *S* value, fault total area *A(t)* value, lack of shock *b* value, statistical *Z* value, and MS entropy *Q*_*t*_ value are all relative indicators, which greatly reduces the errors caused by SOS MS monitoring system problems and increases the accuracy of monitoring and warning information. In general, the warning method of combining the stress-strain curve and multiple MS parameters is more accurate and convincing than the single warning index.

## Conclusions

Multiple MS parameters are proposed to predict coal burst risk combined with the curve of stress vs. strain, and the attenuation rate *K* indirectly verifies that multiple parameters can be effective precursors of coal burst risk. The main conclusions are as follows:

1. The coal burst in LW 1305 was induced by the intrinsic static high-stress concentration and the external strong impact loading generated by fracturing of the key stratum. In detail, the static high-stress concentration was from the deep island face, the igneous rock intrusion and the bifurcation of the coal seam near the damaged region of the coal burst.
2. The MS multiparameter prewarning coal burst risk includes the MS activity *S* value, total fault area $A(t)$ value, lack of shock b value, *Z* value, MS entropy $Q_{t}$ value, and dominant frequency of MS events in the key stratum. In detail, their precursors mainly characterize the enhancement trend of *S*, the sudden and sharp increase in the $A(t)$ value, the continuous and abnormal decrease in the b value, the sharply increasing absolute value of *Z* and greater than 2, the continuous and abnormal decrease in the $Q_{t}$ value, and the dominant frequency moving to the low-frequency band.
3. Considering the time-varying effect of the key stratum, the variations in the multiple MS parameters are strongly correlated with the curve of stress vs. strain, which indicates that it is reasonable to take the observed region as a whole system to research the variation in multiple parameters. The multiple MS parameters should be comprehensively compared to warn of coal burst risk and combined with in situ conditions. It is verified that the accuracy rate of a single monitoring method is very low and cannot meet the actual requirements. More importantly, the multiple MS parameters, stress, drillings volume, reasonable mining arrangement and corresponding prevention measures should be combined to warn and prevent coal burst risk via in situ applications.

## Data Availability

The datasets used and analysed during the current study available from the corresponding author on reasonable request.

## References

[1] Cai W, Dou LM, Si GY, Cao AY, Gong SY, Wang GF, Yuan SS (2019). A new seismic-based strain energy methodology for coal burst forecasting in underground coal mines. Int. J. Rock Mech. Min. Sci..

[2] Li XL, Chen SJ, Wang EY, Li ZH (2021). Rockburst mechanism in coal rock with structural surface and the microseismic (MS) and electromagnetic radiation (EMR) response. Eng. Fail. Anal..

[3] Li ZL, Dou LM, Wang GF, Cai W, He J, Ding YD (2015). Risk evaluation of rock burst through theory of static and dynamic stresses superposition. J. Central South Univ..

[4] Xue RX, Liang ZZ, Xu NW (2021). Rockburst prediction and analysis of activity characteristics within surrounding rock based on microseismic monitoring and numerical simulation. Int. J. Rock Mech. Min. Sci..

[5] Ma TH, Tang CA, Tang LX, Zhang WD, Wang L (2015). Rockburst characteristics and microseismic monitoring of deeply buried tunnels for Jinping II Hydropower Station. Tunn. Undergr. Space Technol..

[6] Gibowicz, S. J. & Kijko, A. An introduction to mining seismology. *Int. Geophys*. (1994).

[7] Brady BT, Leighton FW (1977). Seismicity anomaly prior to a moderate rock burst: A case study. Int. J. Rock Mech. Min. Sci. Geomech. Abstr..

[8] Ma K, Wang HY, Liao ZY, Peng YL, Wang KK (2022). Precursor of microseismic energy and stress evolution induced by rockburst in coal mining: A case study from Xiashijie, Shannxi, China. Geomech. Geophys. Geo-Energy Geo-Resour..

[9] Pailoplee S, Panyatip S, Charusiri P (2017). Precursory seismicity rate changes prior to the large and major earthquakes along the Sagaing fault zone, Central Myanmar. Arab. J. Geosci..

[10] Oynakov E, Botev E (2021). Spatial and time variations of seismicity before strong earthquakes in the southern part of the Balkans. Ann. Geophys..

[11] Marone C, Kilgore B (1993). Scaling of the critical slip distance for seismic faulting with shear strain in fault zones. Nat. Int. Wkly. J. Sci..

[12] Pasten D, Estay R, Comte D, Vallejos J (2015). Multifractal analysis in mining microseismicity and its application to seismic hazard in mine. Int. J. Rock Mech. Min. Sci..

[13] Yang T, Dekkers MJ, Chen JY (2018). Thermal alteration of pyrite to pyrrhotite during earthquakes: New evidence of seismic slip in the rock record. J. Geophys. Res.-Solid Earth.

[14] Zhang EH, Zhou BK, Yang L, Li CF, Li P (2023). Experimental study on the microseismic response characteristics of coal and gas outbursts. Process Saf. Environ. Prot..

[15] Alber M, Fritschen R, Bischoff M, Meier T (2009). Rock mechanical investigations of seismic events in a deep longwall coal mine. Int. J. Rock Mech. Min. Sci..

[16] Manthei. G., Zang, A. & Grosse, C. U. Laboratory acoustic emission in study of rock mechanics. Acoustic Emission Testing, 2 Edn. 477–527 (2022).

[17] Mansurov VA (1994). Acoustic emission from failing rock behavior. Rock Mech. Rock Eng..

[18] Rudajev V, Vilhelm J, Lokajicek T (2000). Laboratory studies of acoustic emission prior to uniaxial compressive rock failure. Int. J. Rock Mech. Min. Sci..

[19] Naoi M, Nakatani M, Otsuki K, Yabe Y, Kgarume T, Murakami O, Masakale T, Ribeiro L, Ward A, Moriya H (2015). Steady activity of microfractures on geological faults loaded by mining stress. Tectonophysics.

[20] Gorgogianni A, Papargyri-Beskou S (2016). Variational analysis of dynamics of fissured poroelastic rocks. Soil Dyn. Earthq. Eng..

[21] Wawersik WR, Fairhurst C (1970). A study of brittle rock fracture in laboratory compression experiments. Int. J. Rock Mech. Min. Sci. Geomech. Abstr..

[22] Manouchehrian A, Cai M (2017). Analysis of rockburst in tunnels subjected to static and dynamic loads. J. Rock Mech. Geotech. Eng..

[23] Feng XJ, Ding Z, Ju YQ, Zhang QM, Ali M (2022). "Double Peak" of dynamic strengths and acoustic emission responses of coal masses under dynamic loading. Nat. Resources Res..

[24] Ortlepp WD, Stacey TR (1994). Rockburst mechanisms in tunnels and shafts. Tunn. Undergr. Space Technol..

[25] Liang ZZ, Xue RX, Xu NW, Li WR (2020). Characterizing rockbursts and analysis on frequency-spectrum evolutionary law of rockburst precursor based on microseismic monitoring. Tunn. Undergr. Space Technol..

[26] Horner RB, Hasegawa HS (1936). The seismotectonics of southern Saskatchewan. Can. J. Earth Sci..

[27] Zhang MW, Shimada H, Sasaoka T, Matsui K, Dou LM (2013). Seismic energy distribution and hazard assessment in underground coal mines using statistical energy analysis. Int. J. Rock Mech. Min. Sci..

[28] Spetzler H, Sondergeld C, Sobolev G, Salow B (1987). Seismic and strain studies on large laboratory rock samples being stressed to failure. Tectonophysics.

[29] Dieterich JH, Smith DE (2009). Nonplanar faults: Mechanics of slip and off-fault damage. Pure Appl. Geophys..

[30] Chai YJ, Dou LM, Cai W, Malkowski P, Li XW, Gong SY, Bai JZ, Cao JR (2023). Experimental investigation into damage and failure process of coal-rock composite structures with different roof lithologies under mining-induced stress loading. Int. J. Rock Mech. Min. Sci..

[31] Hoek E, Martin CD (2014). Fracture initiation and propagation in intact rock-a review. J. Rock Mech. Geotech. Eng..

[32] Hoek E, Brown ET (1997). Practical estimates of rock mass strength. Int. J. Rock Mech. Min. Sci..

[33] Dong LJ, Yan XH, Wang J, Tang Z (2023). Case study of microseismic tomography and multiparameter characteristics under mining disturbances. J. Central South Univ..

[34] Lu CP, Liu GJ, Liu Y, Zhang N, Xue JH, Zhang L (2015). Microseismic multiparameter characteristics of rockburst hazard induced by hard roof fall and high stress concentration. Int. J. Rock Mech. Min. Sci..

[35] Utsu T (1961). A statistical study on the occurrence of aftershocks. Geol. Mag..

[36] Cai W, Dou LM, Zhang M, Cao WZ, Shi JQ, Feng LF (2018). A fuzzy comprehensive evaluation methodology for rock burst forecasting using microseismic monitoring. Tunn. Undergr. Space Technol..

[37] Lu CP, Dou LM, Zhang N, Xue JH, Wang XN, Liu H, Zhang JW (2013). Microseismic frequency-spectrum evolutionary rule of rockburst triggered by roof fall. Int. J. Rock Mech. Min. Sci..

[38] Dou LM, CaiW CAY, Guo WH (2018). Comprehensive early warning of rock burst utilizing microseismic multiparameter indices. Int. J. Min. Sci. Technol..

